# Round the Hospitals

**Published:** 1917-02-10

**Authors:** 


### ROUND THE HOSPITALS.

THOSE nurses and other hospital workers who have had the courage to go right across Europe to Odessa on war work should one day receive a full measure of recognition. We cannot too highly commend Englishwomen for journeying these long distances to nurse the sick. Some of them have laboured assiduously to alleviate the sufferings and restore the wounded Rumanian warriors. This has been done successfully under conditions which often made it exceedingly difficult for the workers to pursue their occupation and maintain their health. The incidence of the war has made constant movement necessary in the case of some workers known to us. They spent a curious Christmas—having travelled all the previous week—Christmas Eve on a barge down the Danube, Christmas Day in an empty barracks, the night in a railway siding, and Boxing Day in the train. Next, a brief space was spent in a Greek Archbishop's house, which provided nothing but a bare shelter, there being a scarcity of wood and no fires anywhere. Fancy such a residence and such conditions for British ladies during the severest of winter weather, faced bravely in the cause of wounded humanity. Instead of repining, these brave British Nurses are looking forward to going to the Front to be attached to a Russian regiment where they are to have an abundance of field work. We are quite sure that there are a large number of our readers who will be glad to have a glimpse of what Miss Margaret Fox, late matron of the Prince of Wales's General Hospital, Tottenham, is doing and how she is faring. She has always been an industrious and consistent worker, and wartime, despite its ever-changing and often most trying conditions, seems to keep her in good health and to satisfy her ambitions. May good fortune attend her and her comrades wherever they may be sent!

MISS MARGARET HOGG, assistant matron, has been elected to the important office of matron to Guy's Hospital, vice Miss L. V. Haughton, whose grievous illness has saddened the whole nursing profession. Miss Hogg has had a wide experience, and has made herself efficient in several fields of work. Trained at Guy's Hospital under Miss Swift, on obtaining her certificate she joined the private nursing staff at Guy's and worked there for nearly two years. She then became first surgical and then medical night sister at Guy's Hospital. Previous to that she took her massage and C.M.B. certificates. In 1911 she was appointed sister to Queen Victoria Ward, and subsequently assistant matron at Guy's Hospital. Her progress, it will be seen, has been rapid, for there are few trained nurses who obtain promotion to the highest post within ten years of the date of their becoming certificated. The fact that Miss Hogg has worked continuously at Guy's Hospital, where the authorities must have had full opportunities to appreciate her technical development, the value of her professional progress, and her administrative gifts, may be taken as conclusive evidence that the new matron is a woman of exceptional energy and capacity. We wish Miss Hogg the best of health, as she will undoubtedly have the whole-hearted co-operation of all workers at Guy's, to enable her to make her mark and fill a most arduous position with infinite credit from the outset, notwithstanding the special difficulties of the moment.

THE Scottish Board of the Royal British College of Nursing, the offices of which are at 122 George Street, Edinburgh, is well on its way to being organised. It promises to be signalised by vigorous management, and we hope it will speedily build up a Register to include all the most competent and fully-trained nurses in Scotland. An important step has been taken in the appointment as secretary of Miss Mary A. Brunton. Miss Brunton, who was trained at Brownlow Hill Infirmary, Liverpool, holds the three years' certificate of that infirmary, where Agnes Jones made a memorable reputation. Miss Brunton has since taken every means to widen her experience and practical knowledge of nursing in all its branches. At the conclusion of her training she became respectively ward sister at the Ear and Throat Hospital, Birmingham, and subsequently at the Blackburn and East Lancashire Infirmary. She then returned to Brownlow Hill Infirmary as assistant superintendent, and left it to take the matronship of the Highfield Infirmary, Liverpool, which she opened and staffed, carrying out the initial work of organising, equipping, and bringing the institution into full activity. She had earlier taken the C.M.B. certificate at Simpson's Memorial Hospital, Edinburgh, and afterwards that of sanitary inspector of the Royal Sanitary Institute. District nursing next claimed her attention, and she joined the Queen's Institute, where she first taught pupils for the C.M.B. certificate, then was appointed, first, inspector of midwives and assistant county superintendent for Surrey, and subsequently county superintendent for Norfolk. Miss Brunton held the latter post at the time of her appointment to the secretaryship of the Scottish Board. She is a native of Scotland, has a reputation for initiative, and has proved her gifts as an organiser and teacher. We wish her and the Scottish Board every success in the important work it will be their privilege to organise for the benefit of the nursing profession.

***** It is our invariable practice to pay for all items of news which we may publish. The minimum payment is 5s. Paragraphs of about twenty lines in length—that is to say, of 150 words or less—are preferred. In this section during the war we propose to give 10s. to the correspondent who may send us in any week the best incident connected with hospital life and work. In this way we hope to afford to all practical aid and encouragement; while those who reciprocate will be able to render us invaluable service. Contributions should be addressed to The Editor, THE HOSPITAL, 28 and 29 Southampton Street, Strand, London, W.C., and, to be in time for the current week's issue, should reach him by the first post on Mondays.**

**For Matrons' and Sisters' Appointments, see page 392.**

